# Size and stochasticity in irrigated social-ecological systems

**DOI:** 10.1038/srep43943

**Published:** 2017-03-07

**Authors:** Arnald Puy, Rachata Muneepeerakul, Andrea L. Balbo

**Affiliations:** 1 Department of Maritime Civilizations, Recanati Institute for Maritime Studies, University of Haifa, 199 Aba Koushy Ave, Mount Carmel, 3498838 Haifa, Israel; 2 Geographisches Institut, Universität zu Köln, Zülphicher Strasse 45, 50674 Cologne, Germany; 3 Department of Agricultural and Biological Engineering, University of Florida, Gainesville, Florida, PO Box 110570, USA; 4 Research Group Climate Change and Security (CLISEC), KlimaCampus, Center for Earth System Research and Sustainability (CEN), University of Hamburg, Grindelberg 5/7, 20144 Hamburg, Germany

## Abstract

This paper presents a systematic study of the relation between the size of irrigation systems and the management of uncertainty. We specifically focus on studying, through a stylized theoretical model, how stochasticity in water availability and taxation interacts with the stochastic behavior of the population within irrigation systems. Our results indicate the existence of two key population thresholds for the sustainability of any irrigation system: 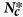 or the critical population size required to keep the irrigation system operative, and N* or the population threshold at which the incentive to work inside the irrigation system equals the incentives to work elsewhere. Crossing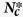 irretrievably leads to system collapse. N* is the population level with a sub-optimal per capita payoff towards which irrigation systems tend to gravitate. When subjected to strong stochasticity in water availability or taxation, irrigation systems might suffer sharp population drops and irreversibly disintegrate into a system collapse, via a mechanism we dub ‘collapse trap’. Our conceptual study establishes the basis for further work aiming at appraising the dynamics between size and stochasticity in irrigation systems, whose understanding is key for devising mitigation and adaptation measures to ensure their sustainability in the face of increasing and inevitable uncertainty.

The discussion on whether the area of irrigation systems is a critical variable for the management of uncertainty cuts through different scientific fields, also involving NGOs and governmental agencies since the end of World War II. The relevance of the debate lies in its implications for the creation and sustainable management of irrigation systems, which currently contribute approximately 40% of the total food produced worldwide and consume approximately 70% of global freshwater supplies^1^.

So far, the discussion has focused on whether ‘small-scale’ or ‘large-scale’ irrigation systems are endowed with better mechanisms for achieving long-term survival in the face of uncertainty, e.g., when exposed to climatic and demographic variability. It has been argued that ‘small-scale’ systems are more able to (1) inhibit the ‘tragedy of the commons’^2^,^3^, (2) minimize conflicts between the top and tail-end irrigators in times of water scarcity^4^, (3) increase the efficacy of internal control institutions^4^,^5^, (4) better cope with pests and market-price fluctuations as a result of the tendency of small-scale farmers to avoid crop specialization^6^,^7^, and (5) reach higher efficiency levels due to the higher degree of collaboration existing in small groups^8^,^9^,^10^,^11^. As for ‘large-scale’ irrigation systems, it has been suggested that they have higher potential for economic profits and rural employment, being able to highly increase welfare and sharply reduce rural poverty and hunger^12^,^13^. Large systems run by centralized organizations might receive higher investments, easing the implementation of modern water-saving technologies and improved crops that help coping with water shortages and population growth. Other authors have suggested that physical dimensions are less relevant for the management of uncertainty than variables such as intensity of land use, land fertility, managerial factors or bureaucratic controls^14^,^15^.

The discussion has thus mostly focused on identifying features or mechanisms characterizing ‘small-scale’ and ‘large-scale’ irrigation systems that might enhance their capacity to cope with climatic or demographic uncertainty. However, it has been shown that climatic and demographic variability also influences the area of irrigation systems: they might shrink or expand following changes in population, water availability or taxation^16^,^17^,^18^,^19^. These variables are intertwined and exert feedbacks on one another: for example, a drop in the volume of water available leading to a reduction of an irrigation system affects its carrying capacity, production output and volume of extractable taxes. An increase in population leading to an enlargement of an irrigated area increases the chances of suffering water shortages and yielding lower outputs per land unit. This shows the extent to which the dynamics between the key size-determining variables might affect the sustainability of irrigation systems. Such systemic behavior, which cannot be appraised by unidirectional approaches tackling one variable at a time, has been overlooked by scientists working on the topic, who have prioritized the accumulation of case studies while pushing the conceptualization of general dynamics into the background. This is a major gap in our knowledge of the sustainability of irrigation systems in the current context of climate change, population growth and political instability, which show large uncertainty ranges due to the complex links between climatic, demographic and geopolitical factors^20^,^21^.

This paper aims at filling this gap. Specifically, it aims at gaining a deeper insight into the dynamics of irrigation systems when disturbed by demographic, environmental or economical variability. Firstly, we present our theoretical framework and discuss the main dynamics between population, water availability and taxation, explaining how they relate to the area of irrigation systems. Secondly, and based on the previous section, we develop a stylized model of population size—which we use as a proxy of the system size—interacting with water availability and taxation. We conclude by discussing the results of the model and their implications for the sustainability of irrigation systems.

## The theoretical framework: stochasticity in size-determining variables

Stochasticity is an essential and inevitable characteristic of social-ecological systems (SESs). Irrigation systems have been frequently studied as SESs^19^,^22^,^23^,^24^,^25^,^26^,^27^,^28^,^29^,^30^. This perspective assumes that the social components of a system (e.g., farmers, water allocation regimes, institutions) are mediated by their interaction with non-cultural biophysical and biological elements (e.g., water resources, flora, soils, climate, topography)^26^,^31^. Social and ecological agents interact at multiple temporal (e.g., years, decades, centuries) and spatial (e.g., local, regional, national) levels and adapt to changing scenarios characterized by variability. As will be elaborated shortly, population, water availability and taxation are three social-ecological variables whose stochasticity plays a key role in determining the size of irrigation systems^16^,^17^,^18^,^19^. If their variability increases within certain thresholds, irrigation systems might remain sustainable without having to modify their area. However, if they change beyond these thresholds, the area will have to be updated or the system may no longer be viable; this might happen when the water available for irrigation drops below the productive needs of the irrigation system. The characterization of the thresholds, or ‘breaking points’, that separate different states of the same social-ecological system, has been the focus of many studies^32^,^33^.

### Population: demographic variance

The size of irrigation systems associated with subsistence agriculture tends to be coherent with the population size managing them^7^,^16^, and higher population density tends to produce larger irrigation systems^34^. The area of irrigation systems is thus highly conditioned by the number of individuals relying on irrigation agriculture.

Population levels evolve randomly due to (1) the random nature of reproduction and death (demographic stochasticity); (2) aleatority in the sex of new members (stochastic sex determination); (3) heterogeneity of the phenotypical characteristics affecting survival and reproduction (demographic heterogeneity); and (4) changes in environmental conditions (environmental stochasticity)^35^,^36^,^37^,^38^,^39^. If any of these sources of demographic variance induces the population size within an irrigation system to drop below a given threshold, de-intensification and abandonment of agricultural land will trigger a reduction in the irrigated area^17^,^40^,^41^. This process is organized in a reinforcing feedback loop: below such a threshold, the per capita labour inputs required to maintain the hydraulic infrastructure (e.g., channels, plots) equal or exceed the per capita payoffs derived from working within the irrigation system. Irrigators then leave and the hydraulic infrastructure degrades, inhibiting the arrival of new users and further downsizing the irrigated area. A process of depopulation and de-intensification has been precisely documented for the irrigation system of Trévelez (South Spain) or the Sikles *khet* (irrigated terraces cropped with wet paddy, Nepal Himalaya), which respectively experienced since 1950 and 1970 a 70% and a 50% reduction due to a massive rural-out migration^17^,^42^.

If population increases beyond such a threshold, the area of the irrigation system might not necessarily need to be enlarged. Users might decide to increase crop production by augmenting the labour input per surface unit, eventually leading to diminished returns^43^. In case users find that additional labour inputs result in zero additional outputs, they might decide to leave the irrigation system to find a better balance between the per capita labour inputs and outputs. When the irrigation system reaches or is close to this point due to a population increase, two options are available in terms of area management: (1) the irrigation area remains the same and some users leave the system moving somewhere else, (2) the irrigation area is enlarged and all users stay. This option needs to concur with a proportional increase in water availability to avoid experiencing water shortages and lower economical returns, which will in turn increase the chances of people leaving the system and triggering the abovementioned reinforcing feedback loop.

### Water availability: environmental stochasticity

The term ‘environmental stochasticity’ designates the randomness in the birth and mortality rates in a given population caused by aleatory fluctuations in external factors^44^, such as rainfall, temperature, or other climatic variables. Rainfall and temperature have dramatic effects on water availability, and thus on the carrying capacity and productive potential of irrigation systems. Given available land, low population pressure and favorable topographical gradients, irrigated areas may be extended or retracted in order to adapt to changes in water availability due to intra and inter annual variations in rainfall. Ebbs and flows in the total area under irrigation due to water variability over the short and the long term have been documented in irrigation systems of Tanzania^18^, Peru^45^, or Turkmenistan^46^. This adaptive response is not as viable in densely populated regions with paucity of arable land and highly dependent on irrigation agriculture, such as Spain. In the Guadalquivir river basin, for instance, a decrease in water resources due to climate change might make the reduction of irrigated areas inevitable in the 21st century^47^,^48^. This will have critical consequences for the regional economy and population welfare, as it will foster a drop in production, economic benefits and the volume of population that the irrigation system can sustain.

In the event of a positive water balance, on the other hand the area of irrigation systems does not automatically need to be enlarged. Although the availability of more water might be a necessary condition for expansion, the reasons behind enlargements are predominantly social rather than climate-driven. Excess water will be used to enlarge an irrigation system when the population depending on such system increases and/or when more produce is deemed necessary, either for consumption, trade or taxation purposes.

### Taxation: political uncertainty

Many states throughout history have largely relied on taxes extracted from irrigated agriculture. Historical examples include the ancient Mesopotamian empires of the third and second millennium BC; the Harappan (India, 2400-1750 BP), the Egyptian Old Kingdom (3100–2181 BC) or al-Andalus (Iberian Peninsula, AD 711–1492), among many others^49^. The volume and intensity of taxes in a given region oscillate not only due to the ephemeral nature of political powers, but also because taxes in irrigation systems are a function of variables showing a random behavior on the long-term, such as population and water availability.

The impact of tax pressure on the area of irrigation systems was originally examined by Barceló^16^,^50^. When no tax-demanding power exists, farmers autonomously decide the area of irrigation systems, which must be sufficient to ensure the survival of the group and yield enough production to buffer unexpected demographic or environmental shocks. When such tax-demanding power exists, irrigators will have to incorporate in their production forecast the need to satisfy its tax demands, and the area of the irrigation system will tend to be larger than it would be otherwise. In any case, both taxes and/or a fraction of the production of the irrigation system must be invested in keeping the irrigation infrastructure in good conditions and ensuring the water flow. The sustainability of any irrigation system in this scenario highly depends on the balance between the extracted taxes/production output and the system’s income: taxation should be enough to keep the infrastructure beyond its maintenance threshold^30^, and still leave some room for the users to benefit from working inside the system. If too high, users might consider the system unprofitable and leave. If too low, the infrastructure might not be properly maintained, and the system will risk collapse.

## Results

It is clear from the preceding sections that an irrigation system is a complex social-ecological system. To gain first-order understanding and establish the basis for future work on this important class of SESs, we developed a stylized model in which stochasticity of population size (*N*), water availability (*W*), and taxation (*T*) interacts. The primary aim of the model is to capture some of the empirical regularities observed above while keeping the model simple enough to yield clear insights. We focus on population since it is the variable that is most closely related to the area of irrigation systems. A detailed description of the model is available in the Methods section, whereas the code can be found as Supplementary Information.

The model is driven by demographic stochasticity and the difference in incentives between working inside and outside the irrigation system,*π(N*; *W, T*)–*π*_*o*_. This latter driving force makes our model akin to a replicator equation. This means that the population size *N* will keep adjusting itself in response to the incentive-based driver while being subject to demographic stochasticity. When the per capita payoff of working inside our focal irrigation system (*π(N*; *W, T*)) is greater than the per capita payoff of these alternative opportunities (*π*_*o*_), *b(N*) > *d(N*) and the population is more likely to increase; if *π(N*; *W, T*) < *π*_*o*_, *b(N*) < *d(N*) and the population is more likely to decrease.

**Figure 1:**
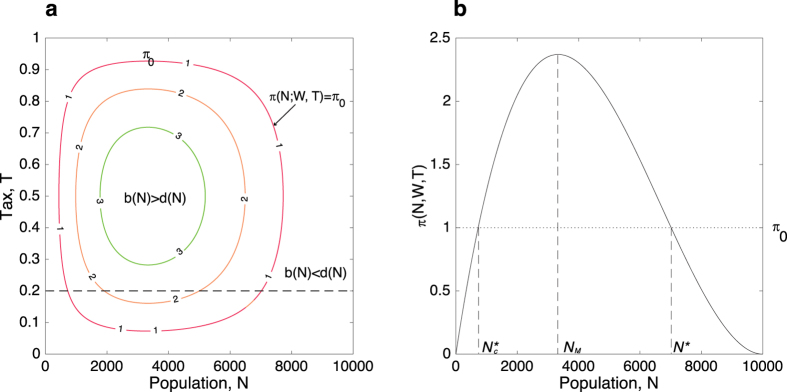
Relationships between the payoff within the irrigation system *π(N*; *W, T*) and population size *N* and tax *T*. (**A**) Contours of *π(N*; *W, T*). Two regions can be identified: more people, on average, entering than exiting the system or *b(N*) > *d(N*) [corresponding to higher payoffs within the irrigation system, *π(N*; *W, T*) > *π*_0_], and more people, on average, exiting than entering the system or *b(N*) < *d(N*) [corresponding to higher payoffs outside the irrigation system, *π(N*; *W, T*) < *π*_0_]. (**B**) *π(N*; *W, T*) as a function of *N* for *T* = 0.2 (intermediate level of sensitivity). For this particular set of parameters (see the Methods section). 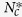 = 727, *N*_*M*_ = 3,333, N* = 7,015.

We can derive a number of important population thresholds from analyzing *π(N*; *W, T*) (Fig. 1a,b): the first one is 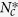, the lower threshold of population size below which irrigators cannot produce and invest enough to maintain the public infrastructure in the long run. 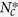 is thus the critical population size for a given irrigation system. The second one is *N*_*M*_, which can be regarded as the population level that yields the maximum value of *π(N*; *W, T*). The third one is N*, the population threshold at which the incentive to work inside the irrigation system equals the incentives to work elsewhere, i.e., *π(N*; *W, T*) = *π*_*o*_. If *π*_*o*_ is consistently greater than the maximum level of *π(N*_*M*_; *W, T*), population will constantly leave the irrigation system and the system will eventually collapse. Figure 1a also shows that N* is the least sensitive to *T* at *T* = 0.5 and increasingly sensitive as *T* increases toward 1 or decreases toward 0. In the following analysis, we used *T* = 0.2, corresponding to an intermediate level of sensitivity.

**Figure 2:**
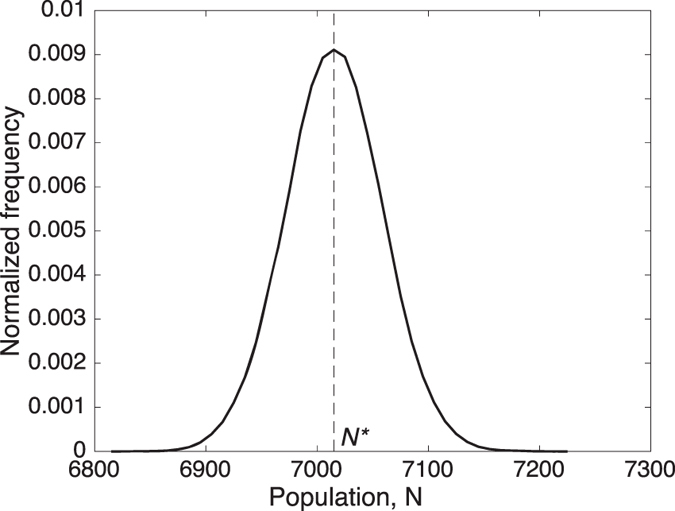
The probability distribution of *N*, calculated from the numerical simulation. N* = 7,015, *CV* = 0.0062.

Figure 2 shows the probability distribution of population size *N* with the same parameters set as in Fig. 1b. This distribution serves as our benchmark to which results that include stochasticity in *W*and *T* will be compared. Due to the shape of *π(N*; *W, T*), there are two levels toward which our system would gravitate: 0 and N*, akin to bistability in deterministic dynamical systems. Such systems possess two basins of attraction and can exhibit a bimodal probability distribution at steady state. In order to have a time series that captures such bimodality, the time series must include a rare series of events that pushes the system from one basin of attraction into another. In this context, for example, it requires that many people randomly decide to leave the irrigation system despite *π(N*; *W, T*) > *π*_0_, a highly improbable (but with non-zero probability) series of events. Rather, our results should be viewed as the probabilistic description of *N* in a ‘short’ timescale, e.g., a timescale that is enough for the system to gravitate toward N* but far shorter than that associated with the highly improbable series of events just mentioned 51. This ‘short’ timescale, we argue, is sufficient and appropriate for addressing the sustainability of the irrigation system.

**Figure 3:**
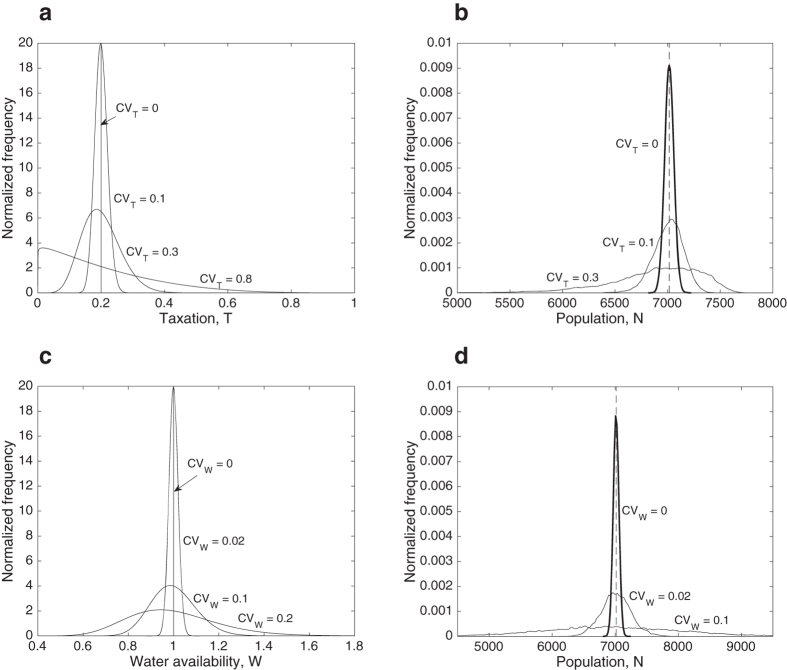
Effects of stochasticity in taxation and water availability. (**A**) Beta distributions associated with the three levels of taxation stochasticity. (**B**) Probability distributions corresponding with the three levels of taxation stochasticity. For *CV*_T_ = 0.8, the irrigation system collapses. (**C**) Log-normal distributions associated with the three levels of water availability stochasticity. (**D**) Probability distributions corresponding with the three levels of water availability stochasticity. For *CV*_W_ = 0.2, the irrigation system collapses.

**Figure 4:**
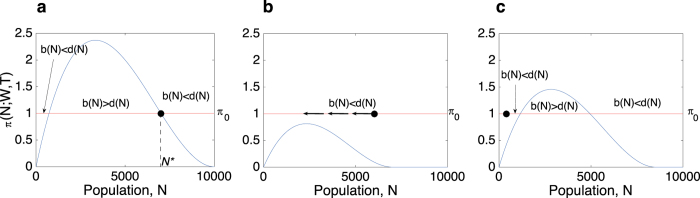
The ‘collapse trap’ mechanism. (**A**) The system has reached the population threshold (N* = 7,015) in which the incentives to remain inside the irrigation system equal the incentives to work elsewhere. (**B**) With strong stochasticity in *W* and *T*, population drops sharply due to *π(N*; *W, T*) < *π*_*o*_. (**C**) Although *π(N*; *W, T*) has increased due to less variability in *W* and *T*, the system is trapped in the region *b(N*) < *d(N*), where individuals are more likely to leave, and cannot readily escape.

Figure 3 shows how stochasticity in taxation and water availability affects population levels. We used three magnitudes of stochasticity for taxation (*CV*_T_ = 0.1, 0.3, and 0.8) and water availability (*CV*_W_ = 0.02, 0.1, and 0.2), with their beta and lognormal distributions shown in Fig. 3a and c, respectively. As water availability and taxation become more uncertain and fluctuate more strongly, so does the population size, with the probability distribution of *N* becoming more skewed to the left and to the right for taxation and water availability, respectively (Fig. 3b and d). When stochasticity in either water availability or taxation is sufficiently strong, e.g., when *W* and *T* take very low and very low/very high values more frequently, population drops sharply. This is because the payoff of working somewhere else exceeds the payoff derived from working inside the irrigation system (Fig. 1a). When this happens, the system heads towards a ‘collapse trap’, even with the stochasticity of *W* and *T* reduced later on. This is because while it is possible to have conditions in which *b(N*) < *d(N*) for all levels of *N*, it is impossible to have conditions in which *b(N*) > *d(N*) for all levels of *N*, given that *π*_*o*_ > 0. Such asymmetry makes such a shift a one-way transition (Fig. 4), hence a ‘trap’.

## Discussion and Conclusions

Our results suggest the existence of two important population thresholds that should be taken into account when considering the sustainability of any irrigation system: 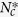 and N* (Fig. 1b). If population decreases below 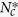 or exceeds N*, the payoff of working within the irrigation system is smaller than the payoff of working elsewhere, leading to declines in population. This mechanism is triggered by the structure of *π(N*; *W, T*) and is specially critical for irrigation systems with a population size slightly beyond 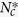. In this context, any drop in population due to demographic stochasticity risks putting the system in the region *π(N*; *W, T*) < *π*_*o*_. Once the population falls below 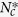, the system is at great risk of an irreversible decline toward a collapse. For irrigation systems with a population size slightly below N*, surpassing this threshold due to a population increase caused by demographic stochasticity or immigration will not be as deleterious: the system will readjust itself with individuals, on average, leaving the irrigation system until the population returns to levels around N*, where the difference in incentives between working inside and working outside the irrigation system disappears.

These system behaviors raise two relevant issues. The first one is that irrigation systems with populations close to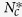 are more prone to collapse simply due to aleatory fluctuations in the number of births and deaths. Therefore, aiming for more offspring than is necessary, or simply aiming at attracting more individuals, may be seen as a strategy to avoid collapse by groups of irrigators as it positions the system further away from this critical threshold. This idea is in agreement with the study by Winterhalder and Leslie^52^ on fertility and risk among smallholders. By conceptualizing the relationship between fertility and fitness as an S-shaped function, in which each sibling adds significantly to the family performance until an inflection point is reached, Winterhalder and Leslie^52^ observed that having fewer-than-optimum offspring is riskier for the family unit than ending up with a larger-than-optimum cohort. This observation may be couched in the context of our analysis. At N* > *N*_*M*_ (“larger than optimum”), the per capita payoff of working in the irrigation system is lower than its maximum value achieved at *N*_*M*_, thereby incurring some sub-optimal performance. However, at *N* closer to 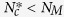 (“fewer than optimum”), in addition to the per capita payoff of working in the irrigated system being lower than its maximum value, the system is also at risk of an irreversible collapse. The crucial role of child-labour in smallholder economy^7^,^53^, along with the attempts to minimize the harmful effects of demographic variance, could thus partially explain the high fertility rate detected by some authors, such as Bentley *et al*.^54^,^55^ or Sellen and Mace^56^, in some intensive agricultural societies. This strategy, e.g., aiming at a larger population than the minimum required, resonates within our model as a mechanism to avoid collapse in irrigation systems.

The second relevant issue is that, on a larger time-scale, irrigation systems without any mechanism to regulate population inputs would gravitate, in a self-organized fashion, towards achieving their “equilibrium” population size N* at which incentives of working inside and outside of the irrigation system are equal. In other words, as long as the payoffs of working inside exceed those derived from working outside the system, population will likely keep increasing. Now, recall that at N*, the per capita payoff is sub-optimal. But per the discussion above, N* is a stable and resilient state at a relatively low level of wealth, a situation with some similarity to an open-access outcome or a ‘poverty trap’^57^. If maintaining a high per capita payoff is a goal (e.g., keeping the system close to *N*_*M*_), some institutional arrangement will be needed that controls well-defined population boundaries beyond which no access to the resource is granted. This is related to the first principle Ostrom proposed as key for the long-term sustainability of the commons, e.g., individuals or households who have rights to withdraw resource units from the common-pool resource must be clearly defined, as must the boundaries of the common-pool resource itself^5^.

Although processes leading to the collapse of different SES have been widely described in the literature (see for instance the special feature Critical Perspectives on Historical Collapse, *PNAS* 2012), many have been framed within the adaptive cycle of Holling^58^,^59^,^60^,^61^,^62^. In this framework, collapse is sometimes followed by system reorganization, with the system either remaining within the same regime or autonomously changing to a new one with the same or different state variables^63^. Our results indicate that, when the stochastic dynamics of the size-determining variables are accounted for, the self-reorganization of irrigated SESs after collapse might be impossible. The ‘collapse trap’ mechanism suggests that they are irreversibly bound to fail under persistent strong climatic and taxation variability—unless some measures for counteracting population drops within the irrigation system are swiftly implemented before the system crosses 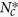. Although proposing possible policies is beyond the scope of this paper, such measures should take into account that population drops are motivated by *π(N*; *W, T*) < *π*_*o*_, i.e., they are driven by the existence of higher economic incentives out of the irrigation system. This forces any agenda to consider the larger socio-economical context within which the irrigation system is nested and apply managerial practices simultaneously at different scales^64^. Once the system crosses 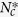, the ‘collapse trap’ mechanism also explains why large external injections of assistance (e.g., capital inputs, subsidy for infrastructure maintenance, or modernization by means of new irrigation technologies) might be necessary to move abandoned irrigation systems out of the *π(N*; *W, T*) < *π*_*o*_ zone.

The above discussion helps sharpen the ‘size’ debate. The key question for sustainability is not so much whether ‘small-scale’ (e.g., between 1–100 ha, see FAO^65^) or ‘large-scale’ (e.g., larger than 2000 ha, see Vincent^66^) irrigation systems better cope with uncertainty, but rather how close an irrigation system is to either N* or 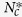 and how strong the stochasticity in the size-determining variables is. Importantly, both ‘small-scale’ and ‘large-scale’ irrigation systems can potentially collapse under sufficiently strong variability in either water availability or taxation. With the size of irrigation systems being an emergent property in response to population, water availability and taxation^67^, it is the interaction between those variables and their degree of stochasticity that determine whether an irrigation system is ‘too large’ (e.g., too close to N*) or ‘too small’ (e.g., too close to 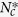) to be sustainable. If only demographic stochasticity is accounted for, our results thus suggest that being ‘too small’ is much more threatening than being ‘too large’ for the long-term sustainability of irrigation systems.

In this paper, we show that explicitly and dynamically incorporating stochasticity of variables that play important roles in determining the size of irrigation systems yields novel insights that one would not gain otherwise. The theoretical framework and the modeling approach are promising as they are in agreement with empirical regularities observed in irrigation systems. This work also serves as a conceptual basis to guide further research on the topic. Fieldwork and the development of case studies following this conceptualization can provide more nuanced insights and can eventually lead to modification of the model structure and its underlying theory. It is also worth noting that we exclusively focused the model on analyzing the effects that stochasticity in water availability and taxation has—in isolation—on the population size. A natural extension of our work is to consider cases in which fluctuations in water availability and taxation are dependent and/or with certain temporal structure. Other potential directions include specifying mechanistic dynamics of water availability and taxation. These will likely yield additional critical thresholds related to the sustainability of irrigation systems and ultimately deeper understanding of the complexity of these important systems and better epistemological tools to avert their collapses.

## Methods

A model is developed to capture the interplay between population and the two other stochastic variables conditioning the area of irrigation systems, water availability and taxation. To capture population stochasticity we treat population size *N* as a random variable, and select a small time step *dt* such that *N* may increase or decrease by at most 1. The rate at which *N* increases during this time step is *b(N)dt*, and the rate at which it decreases is *d(N)dt*. The probability that *N* increases to *N* + 1 in this time step is *b(N*)/(*b(N*) + *d(N*)) and the probability that *N* decreases to *N* − 1 in this time step is *d(N*)/(*b(N*) + *d(N*)).

To capture the underlying forces that cause individuals to enter or leave the system, we specify


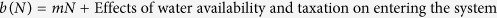



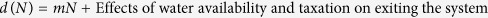


where *m* represents birth and death as well as immigration and emigration to and from the irrigation system.

We now focus on capturing the ‘effects of water availability and taxation’ on *b(N*) and *d(N*). We frame these effects in the form of incentive compared to other opportunities in the region, be they employment in regional urban areas or other nearby irrigation systems. Let *π*_*o*_ denote the average *per capita* payoff of these alternative outside opportunities. As such, the ‘effects of water availability and taxation’ can be expressed as the difference between the *per capita* payoff of working inside our focal irrigated system, *π(N*; *W, T*), and *π*_*o*_—with *π(N*; *W, T*), being a function of water availability *W*, taxation level *T*, and the population size. If *π(N*; *W, T*) > *π*_*o*_, *b(N*) will increase; if *π(N*; *W, T*) < *π*_*o*_, *d(N*) will increase.

In particular, if


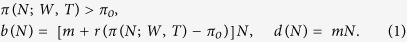


And if


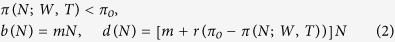


where *r* is the responsiveness of the population to the payoff incentive.

We now focus on specifying *π(N*; *W, T*). The two elements to which we will pay close attention during this specification are the roles of infrastructure and feedbacks. Infrastructure needs sufficient maintenance, or it will otherwise degrade and eventually collapse. Infrastructure in good conditions enables the population to make use of the resource—water, in this case. Assuming that the population is required to pay a fraction *T* of the income they derive from water as tax, we may write


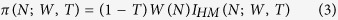


where *I*_*HM*_(*N*; *W, T*) represents the influence of the human-made (hence the subscript HM) infrastructure on the ability of individuals to make use of the available water *W*. We have made *W* a function of *N*: as more people use the water, less water would become available. Here we use the following simple linear relationship to capture this:


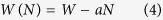


where *a* represents *per capita* usage rate of the population. This relationship already imposes a constraint on *N*: If *N* ≥ *W/a, W* = 0 (one cannot have a negative amount of water) and *π(N*; *W, T*) = 0. We have also made *I*_*HM*_ a function of *N* and *T* as we assume that the more people make use of the water and the more tax they pay, the better condition the infrastructure would be in and the more water each individual can extract. This provides the feedback from the population size and taxation to *π(N*; *W, T*), which in turn affects the rates at which people enter or leave the system. From these assumptions, we write


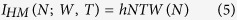


where *h* captures the level of income each individual makes from a unit of available water. Inserting these expressions back into *π(N*; *W, T*), we arrive at


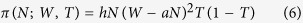


Through embedding the feedbacks among income, taxation, and the ability to harvest water through infrastructure in the incentive for an individual to enter or leave the system, we have derived the above nonlinear relationship between these factors. It should be noted that other, more nuanced feedbacks are possible: for example, the state of infrastructure *I*_*HM*_ may influence how *W* and *N* interact, thereby changing the nature of function *W(N*). Such additional feedbacks can be built upon a solid, simple model we are building.

Putting all these elements together, we have the following model.

At each time step, if


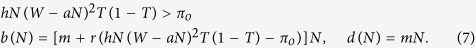


If


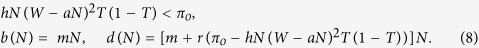


Finally, at the boundary condition, when *N* = 0, *b*(0) = *m* and *d*(0) = 0.

Since demographic stochasticity is intrinsic in our model, we first used the probabilistic description of *N* with *W* and *T* fixed as the benchmark. We then introduced stochasticity in *W* and *T* to examine how they interacted with the intrinsic demographic stochasticity. As *N* changes by at most 1 during each time step *dt*, the time step must be interpreted as quite small. Therefore, it is more reasonable to assume that *W* and *T* fluctuate at a larger time scale. To make this assumption concrete, we assumed that *dt* = 0.00005 year (approximately 26 minutes) and that the values of *W* and *T* would change only once a year, i.e., 20,000 time steps.

For *W*, we used a log-normal distribution for its fluctuation, as only positive values make sense. For *T*, we used a beta distribution for its fluctuation, as it can only take on a value in a finite range [0, 1]. Since we are interested in the effects of stochasticity, in prescribing the uncertainty of *W* and *T* we kept their mean values fixed and changed only their variances. To better put the magnitude of variability in context, we report them as coefficients of variation (*CV*), e.g., the standard deviation normalized by the mean.

The values of the parameters used in the model are as follows: *m* = 1, *h* = 0.01, *r* = 1, *a* = 0.0001. The means of *W* and *T* are fixed at 1 and 0.2, respectively.

The full code is provided as Supplementary Information.

## Additional Information

**How to cite this article**: Puy, A. *et al*. Size and stochasticity in irrigated social-ecological systems. *Sci. Rep.* **7**, 43943; doi: 10.1038/srep43943 (2017).

**Publisher's note:** Springer Nature remains neutral with regard to jurisdictional claims in published maps and institutional affiliations.

## Supplementary Material

Supplementary Information
