# Supplementary material for: Size and stochasticity in irrigated social-ecological systems
Source: Sci Rep. 2017 Mar 7;7:43943. doi: 10.1038/srep43943 (PMC5339736; doi:10.1038/srep43943)
Supplement: Supplementary Information [file srep43943-s1.pdf]

Size and stochasticity in irrigated social-ecological systems

Supplementary Information

Arnald Puy, Rachata Muneepeerakul, Andrea L. Balbo

```
% Matlab code of the model simulation
clear, clc
format compact
rng('shuffle')

% Parameter values ---
m = 1
r = 1
h = 0.01
a = 0.0001
mW = 1 % mean W
mT = 0.2 % mean T
% --------------------

% Prescribing levels of stochasticity ---
cvW = 0 % 0, 0.02, 0.1, 0.2
cvT = 0.1 % 0, 0.1, 0.3, 0.8
% Prescribing levels of stochasticity ---

% Statisitcs of stochastic W
vW = (cvW*mW)^2
mu = log((mW^2)/sqrt(vW+mW^2));
sigma = sqrt(log(vW/(mW^2)+1));

% Statistics of stochastic T
vT = (cvT*mT)^2
aa = (mT*(1-mT)/vT-1)*mT;
bb = (mT*(1-mT)/vT-1)*(1-mT);

%% Plot relationship between population and payoff at mean W and mean T
W = mW;
T = mT;
hT1T = h*T*(1-T)
K = W/a;
pio = 1
Wmin = 3*(a*pio/(4*h*T*(1-T)))^(1/3)

figure(1)
X = 0:round(W/a);
Y = hT1T*X.*(W-a*X).^2;
plot(X,Y,'b')
hold on
plot([0 max(X)],[1 1]*pio,'r')
ax = gca;
ax.FontSize = 30;
xlabel('Population level, N')
ylabel('\pi(N;W,T)')
text(10050,1,'\pi_0','fontsize',28)

Nstar = fzero(@(x) hT1T*x.*(W-a*x).^2-pio, 7000)

%% Run simulation
countYr = 0;
nYear = 2000
annual = 20000
timeHorizon = round(nYear*annual)
Nvec = zeros(timeHorizon,1);
Nvec(1) = round(Nstar);
Wvec = zeros(nYear,1);
Tvec = zeros(nYear,1);

for t = 1:timeHorizon-1
    if mod(t-1,annual)==0
```

```
        countYr = countYr + 1;
        if vW>0
            W = lognrnd(mu,sigma); % stochastic W
        else
            W = mW;
        end
        if vT>0
            T = betarnd(aa,bb); % stochastic T
        else
            T = mT;
        end
        Wvec(countYr) = W;
        Tvec(countYr) = T;
        K = W/a;
        hT1T = h*T*(1-T);
    end
    N = Nvec(t); % if N==0, figure, plot(Nvec), return, end

    if N >= K
        piN = 0;
    else
        piN = hT1T*N*(W-a*N)^2;
    end
    if piN>=pio
        bN = (m+r*(piN-pio))*N;
        dN = m*N;
    else
        bN = m*N;
        dN = (m+r*(pio-piN))*N;
    end
    if N==0
        bN = m;
        dN = 0;
    end

    randTable = [bN/(bN+dN) 1];
    dummy = rand;
    for j = 1:2
        if dummy<=randTable(j)
            break;
        end
    end

    switch j
        case 1
            Nvec(t+1) = N+1;
        case 2
            Nvec(t+1) = N-1;
    end
end

%% Plot the probability density function of population size
figure(2)
Nms = Nvec(min(find(Nvec>Nstar)):timeHorizon); % ms = metastable
binSiz = 20;
halfBin = binSiz/2;
[F,bin] = hist(Nms,[min(Nms)-halfBin:binSiz:max(Nms)+halfBin]);
relFreq = F/length(Nms)/binSiz;
plot(bin,relFreq,'k')
hold on
plot([Nstar Nstar],[0 0.01],'--k')
ax = gca;
ax.FontSize = 18;
```

```
xlabel('Population size, N')
ylabel('Normalized frequency')
meanN = mean(Nms)
stdN = std(Nms)
varN = var(Nms)
skewN = skewness(Nms)
cvN = stdN/meanN
```
